# PAPP-A-Specific IGFBP-4 Proteolysis in Human Induced Pluripotent Stem Cell-Derived Cardiomyocytes

**DOI:** 10.3390/ijms24098420

**Published:** 2023-05-08

**Authors:** Daria A. Adasheva, Olga S. Lebedeva, Daria V. Goliusova, Alexander B. Postnikov, Maria V. Teriakova, Irina V. Kopylova, Maria A. Lagarkova, Alexey G. Katrukha, Daria V. Serebryanaya

**Affiliations:** 1 Department of Biochemistry, Faculty of Biology, Lomonosov Moscow State University, 119991 Moscow, Russia; 2 Lopukhin Federal Research and Clinical Center of Physical-Chemical Medicine of Federal Medical Biological Agency, 119435 Moscow, Russia; 3 Hytest Ltd., 20520 Turku, Finland

**Keywords:** cardiomyocytes, proteolysis, PAPP-A, IGF, IGFBP-4

## Abstract

The insulin-like growth factors IGF-I and IGF-II—as well as their binding proteins (IGFBPs), which regulate their bioavailability—are involved in many pathological and physiological processes in cardiac tissue. Pregnancy-associated plasma protein A (PAPP-A) is a metalloprotease that preferentially cleaves IGFBP-4, releasing IGF and activating its biological activity. Previous studies have shown that PAPP-A-specific IGFBP-4 proteolysis is involved in the pathogenesis of cardiovascular diseases, such as ischemia, heart failure, and acute coronary syndrome. However, it remains unclear whether PAPP-A-specific IGFBP-4 proteolysis participates in human normal cardiomyocytes. Here, we report PAPP-A-specific IGFBP-4 proteolysis occurring in human cardiomyocytes derived from two independent induced pluripotent cell lines (hiPSC-CMs), detected both on the cell surface and in the cell secretome. PAPP-A was measured by fluoroimmune analysis (FIA) in a conditioned medium of hiPSC-CMs and was detected in concentrations of up to 4.3 ± 1.33 ng/mL and 3.8 ± 1.1 ng/mL. The level of PAPP-A-specific IGFBP-4 proteolysis was determined as the concentration of NT-IGFBP-4 proteolytic fragments using FIA for a proteolytic neo-epitope-specific assay. We showed that PAPP-A-specific IGFBP-4 proteolysis is IGF-dependent and inhibited by EDTA and 1,10-phenanthroline. Therefore, it may be concluded that PAPP-A-specific IGFBP-4 proteolysis functions in human normal cardiomyocytes, and hiPSC-CMs contain membrane-bound and secreted forms of proteolytically active PAPP-A.

## 1. Introduction

The insulin-like growth factors IGF-I and IGF-II mediate a wide range of physiological and pathological processes in cardiac tissue, including growth promotion, cardiac contractility improvement, tissue remodeling, metabolic regulation, cell regeneration, hypertrophy stimulation, autophagy, and apoptosis [1]. Additionally, it has been demonstrated that the IGFs, particularly IGF-I, exert cardioprotective effects against myocardial injury and can restore heart function after infarction [2,3]. These findings imply that IGFs may be crucial for maintaining cardiomyocyte viability in healthy cardiac tissue; however, the mechanism underlying IGF bioavailability in the healthy heart is far from being completely understood.

The bioavailability of IGFs in various cells and tissues is essentially regulated by two groups of proteins: IGFBP proteins (insulin-like growth factor binding proteins 1–6), which form a complex with IGFs and inhibit their activity; and proteases, which specifically cleave IGFBP in the IGF-IGFBP complex, leading to IGF release. This process enables the factors to interact with specific receptors and trigger the appropriate signaling pathways [4]. One of the IGFBP proteins, IGFBP-4, is known to be specifically cleaved by protease PAPP-A (pregnancy associated plasma protein A), a zinc metalloprotease associated with the extracellular side of the cell membrane [5]. PAPP-A exists in two forms: a dimeric proteolytically active form, which is associated with the cell surface through glycosaminoglycan (GAG) [6]; and a proteolytically inactive heterotetrameric PAPP-A form, consisting of dimeric PAPP-A and two subunits of the pro-form of eosinophil major basic protein (proMBP) [7]. In the case of IGFBP-4, PAPP-A is the only protease that cleaves this protein in physiological conditions. The PAPP-A-specific proteolysis of IGFBP-4 occurs at a specific site localized between Met135 and Lys136. As a result of the cleavage, two proteolytic fragments of the protein are formed: N-terminal (NT-IGFBP-4) and C-terminal (CT-IGFBP-4) [8]. Previous studies have shown that PAPP-A cleaves IGFBP-4 in the conditioned medium of fibroblasts and follicular fluid of human, bovine, porcine, and horse ovaries [9,10]. Many cell types, including fibroblasts [11], granulosa cells [12], vascular smooth muscle cells [13], endometrial stromal cells [14], cardiac adipocytes [15], endothelial cells and smooth muscle cells of the human coronary artery [16], and progenitor cells of the human heart [17], secrete PAPP-A.

It was previously demonstrated that PAPP-A is involved in the development of pathological states of the heart [5,18,19,20,21,22,23]. PAPP-A-derived IGFBP-4 proteolytic fragments are considered to be diagnostic and prognostic biomarkers of various cardiovascular diseases (CVDs), such as acute coronary syndrome (ACS) [18], acute heart failure [19], and ischemia [20]. However, it remains unknown whether PAPP-A-specific IGFBP-4 proteolysis occurs in the normal heart. Although our group reported that PAPP-A-specific proteolytic IGFBP-4 cleavage occurs in the primary culture of rat neonatal cardiomyocytes [21], there is no evidence for PAPP-A-dependent IGFBP-4 proteolysis in human normal cardiomyocytes.

hiPSC-CMs have been shown to be a promising model for investigating normal and pathological cardiac processes [24,25,26,27,28,29,30]. hiPSC-CMs are similar to adult cardiomyocytes in many aspects, as they express most of the components that mediate excitation–contraction coupling and membrane voltage regulation. hiPSC-CMs also express different types of ion channels required for the depolarization and repolarization of the membranes, structures of sarcoplasmic reticulum calcium release and uptake [24,27], and especially members of signal cascades typical of adult cardiomyocytes [25,31]. Accordingly, we applied human hiPSC-CMs as a model to investigate PAPP-A-specific IGFBP-4 cleavage in human cardiomyocytes in a normal state and described the specificity and localization of PAPP-A-dependent IGFBP-4 proteolysis.

## 2. Results

### 2.1. Generation and Characterization of Human iPSC-Derived Cardiomyocytes

**Figure 1:**
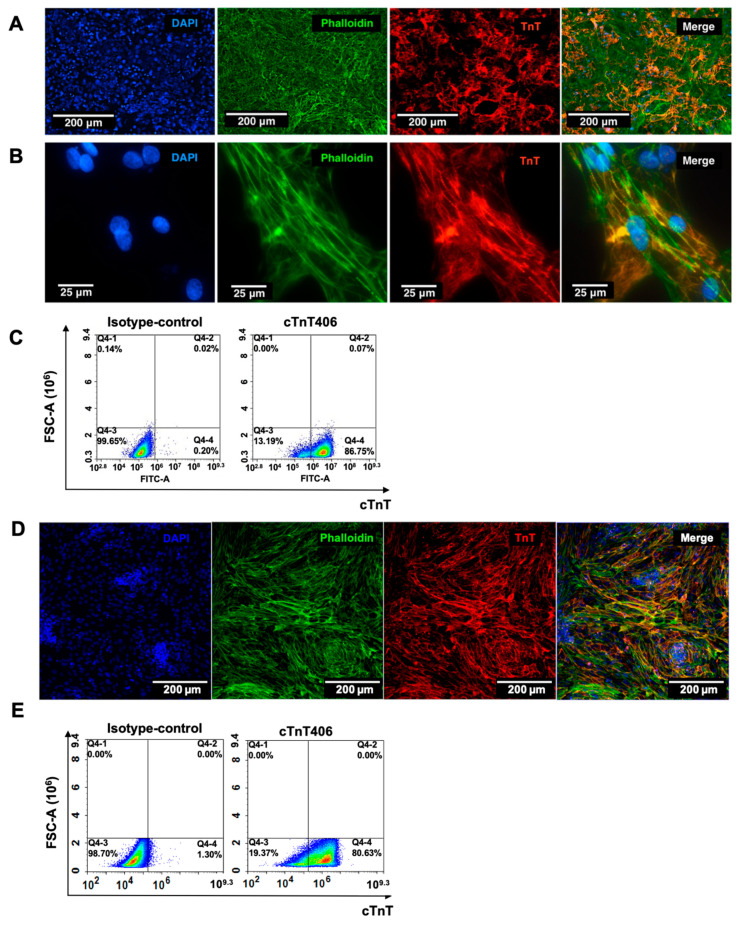
Characterization of the hiPSC-CM cultures. (**A**–**C**) hiPSC-CMs derived from endo-IPS12 cell line; (**D**,**E**) hiPSC-CMs derived from RG6L cell line. (**A**,**B**,**D**) Immunofluorescent staining of hiPSC-CMs using (left to right): DAPI—blue; phalloidin—green; and human cardiac troponin T isoform—red; and overlay of images obtained in three fluorescent channels at the same magnification. (**A**,**D**) Scale bar 200 µm. (**B**) Scale bar 25 µm. (**C**,**E**) Representative flow cytometry analysis of hiPSC-CMs. Left—hiPSC-CMs stained with isotype-control antibody. Right—hiPSC-CMs stained with antibodies specific to cardiac marker cTnT.

We applied a human iPSC-CM model to study PAPP-A-specific IGFBP-4 proteolysis in normal human cardiomyocytes. Both PAPP-A and IGFBP-4 appear to be members of the IGF signaling system and do not participate in depolarization/repolarization and contraction events; therefore, the hiPSC-CM model can be utilized to study PAPP-A-specific IGFBP-4 proteolysis. Cardiomyocytes were differentiated from the hiPSC-lines endo-IPS12 and RG6L as described in Section 4. After 18 days of differentiation, the cultivation of cardiomyocytes in the maintenance medium was prolonged to 32 days, as several studies have reported that the prolonged cultivation of hiPSC-CMs could improve their maturity in morphology, structure, and physiology [32,33]. The resulting populations of hiPSC-CMs differentiated from both cell lines on the 50th day of differentiation displayed contraction rates of 22 and 28 beats per minute for endo-IPS12 and RG6L-derived hiPSC-CMs, respectively (see Supplementary Videos). The efficiency of differentiation was evaluated by immunofluorescence staining (Figure 1A,B,D) and flow cytometry analysis (Figure 1C,E). The cells on the 50th day of differentiation were stained with antibodies specific to the cardiac marker troponin T (cTnT), phalloidin for actin detection, and DAPI (Figure 1A,D). It could be clearly seen that the cytoskeleton of hiPSC-CMs was striated, indicating the sufficient maturity of cardiomyocytes (Figure 1B).

The purity of the resulting cardiomyocyte populations was calculated as the ratio of cells with detected troponin T to the total number of cells, corresponding to 90 ± 2%. Differentiation efficiency was also determined by flow cytometry analysis, corresponding to 87% and 91% in the cases of endo-IPS12 and RG6L-derived cardiomyocytes, respectively (Figure 1C,E). Thus, highly pure, characterized populations of contracting cardiomyocytes were used to study PAPP-A specific-IGFBP-4 proteolysis.

### 2.2. IGFBP-4 Is Proteolytically Cleaved by PAPP-A in hiPSC-CM-Conditioned Medium

**Figure 2:**
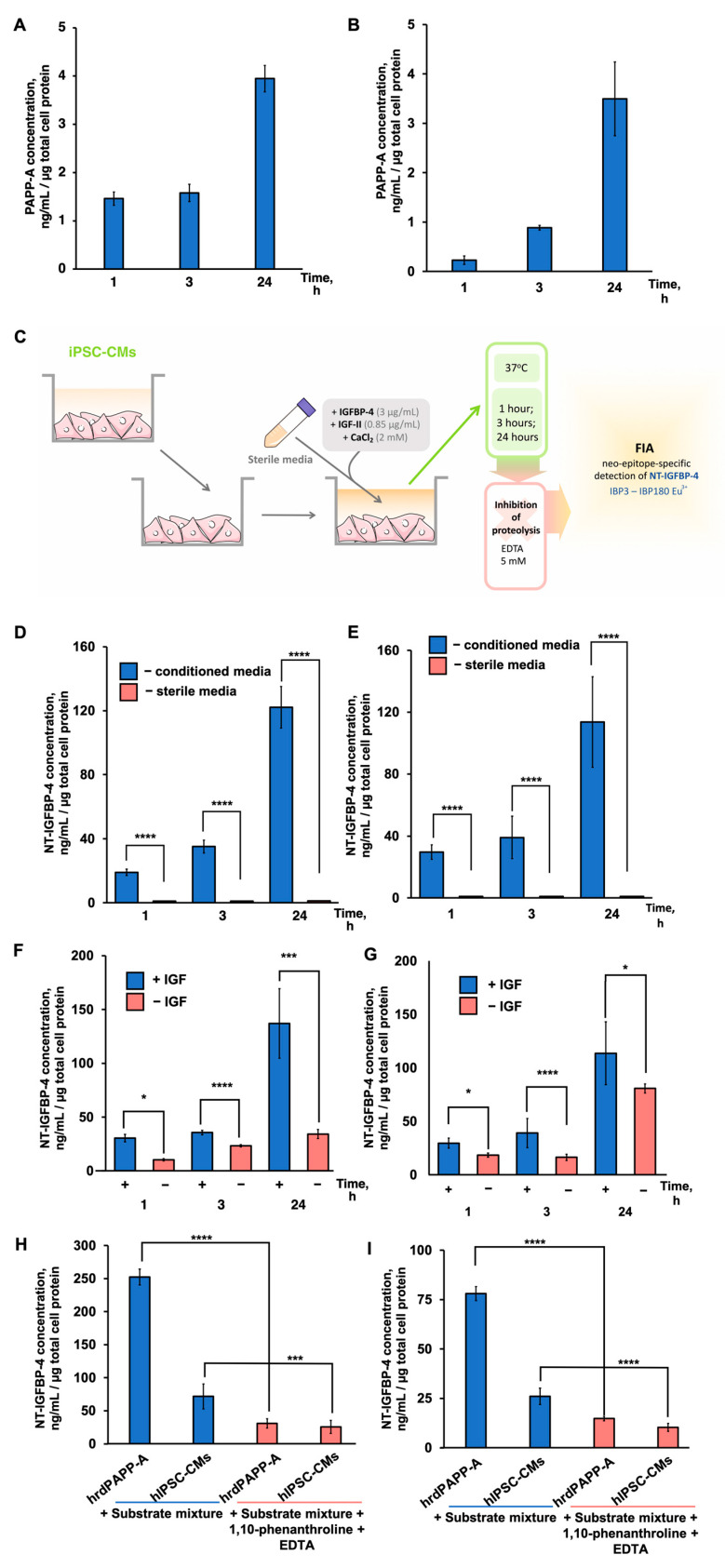
PAPP-A-specific IGFBP-4 cleavage in hiPSC-CMs. (**A**,**D,F,H**) endoIPS-12-derived hiPSC-CMs. (**B**,**E**,**G**,**I**) RG6L-derived hiPSC-CMs. (**A**,**B**) Measurement of PAPP-A concentration in hiPSC-CM-conditioned medium. The medium was changed to a fresh medium, and the PAPP-A concentration was measured after 1, 3, and 24 h of cultivation, as described in Section 4; *n* = 3. (**C**) Experimental design to study the PAPP-A-specific IGFBP-4 proteolysis in iPSC-CMs. (**D**,**E**) Time course of NT-IGFBP-4 accumulation after 1, 3, and 24 h of IGFBP-4 proteolytic reaction (*n* = 11, **** *p* < 0.0001). (**F**,**G**) Effect of IGF-II on accumulation of NT-IGFBP-4 in the process of proteolytic cleavage of IGFBP-4 in the conditioned medium of hiPSC-CMs (*n* = 4, **** *p* < 0.0001, *** *p* < 0.001, * *p* < 0.05). (**H**,**I**) PAPP-A-dependent IGFBP-4 proteolysis is inhibited by ethylendiaminetetraacetic acid (EDTA) and 1,10-phenanthroline in an in vitro proteolytic reaction and in hiPSC-CM culture media (*n* = 4, **** *p* < 0.0001, *** *p* < 0.001). hrdPAPP-A—human recombinant dimeric PAPP-A. The obtained concentrations were normalized to total protein content measured by Bradford protein assay (see Section 4).

We measured the PAPP-A concentrations in the conditioned medium (cell secretome) of hiPSC-CMs by 10E1–10E2 assay, which recognizes both homodimeric and heterotetrameric PAPP-A. PAPP-A was accumulated in the conditioned medium of endo-IPS12- and RG6L-derived hiPSC-CMs within 24 h of hiPSC-CM cultivation at concentrations of up to 4.3 ± 0.3 ng/mL and 3.8 ± 1.1 ng/mL, respectively. (Figure 2A,B). The levels of tetrameric PAPP-A detected in the endo-IPS12- and RG6L-derived hiPSC-CM-conditioned medium using a 7A6–5H9 assay were below the detection level of the assay (0.05 ng/mL). Thus, we suggested that all detected PAPP-A was homodimeric. Calculated PAPP-A concentrations were comparable to concentrations measured for PAPP-A secreted by fibroblasts [11,34] and coronary artery smooth muscle cells [16,35], although there are some limitations concerning different types of assays applied for concentration measurements. If compared to cardiac progenitor cells, the concentrations of PAPP-A measured in hiPSC-CM-conditioned medium were significantly higher [17]. Thus, it was observed that hiPSC-CMs secreted a homodimeric form of PAPP-A into the conditioned medium.

We previously demonstrated that PAPP-A-specific IGFBP-4 proteolysis occurs in the conditioned medium of neonatal rat primary culture cardiomyocytes [21], although there was no evidence that PAPP-A-specific IGFBP-4 proteolysis occurred in the conditioned medium of normal human cardiomyocytes. We performed the IGFBP-4 proteolytic reaction in the conditioned medium of hiPSC-CMs, as shown in Figure 2C. The concentration of NT-IGFBP-4 in hiPSC-CM-conditioned medium was measured by FlA using the antibody combination IBP3-IBP180-Eu^3+^ specific to the proteolytic neo-epitope. This epitope is formed as a result of specific IGFBP-4 proteolysis by PAPP-A. As shown in Figure 2D,E, NT-IGFBP-4 accumulated in the conditioned medium of hiPSC-CMs derived from endo-IPS12 and RG6L cell lines after 1, 3, and 24 h at concentrations that corresponded to 19.0 ± 1.3, 35.0 ± 2.5, and 122.15 ± 5.3 ng/mL and 29.0 ± 2.3, 42.0 ± 1.5, and 113.55 ± 4.3 ng/mL, respectively, while no NT-IGFBP-4 was observed in sterile medium. NT-IGFBP-4 was also detected at 170.55 ± 4.3 ng/mL levels when IGFBP-4 proteolysis was performed for 24 h using exogenously added recombinant PAPP-A in sterile medium to show that its proteolytic activity was sustained in conditions of hiPSC-CM cultivation.

As previously shown, IGF enhances PAPP-A-specific IGFBP-4 proteolysis, as PAPP-A is capable of cleaving IGFBP-4 more efficiently when IGFBP-4 forms a complex with IGF [36]. Figure 2F,G shows the time course of NT-IGFBP-4 formation in hiPSC-CM-conditioned medium in the presence and absence of IGF-II. In both cell lines in the presence of IGF-II, the concentrations of NT-IGFBP-4 in conditioned hiPSC-CM medium were 1.5–3 times higher than in the absence of IGF-II, indicating that IGF-II was required for the proteolytic cleavage of IGFBP-4 in hiPSC-CM-conditioned medium.

It was reported that PAPP-A is a metalloproteinase that contains Zn^2+^ in the active center, and it requires both Zn^2+^ and Ca^2+^ for its proteolytic activity [37]. To test whether IGFBP-4 proteolysis in a hiPSC-CM-conditioned medium is dependent on the presence of Ca^2+^and Zn^2+^, the hiPSC-CMs and human recombinant homodimeric PAPP-A, used as control, were pretreated with the classical metalloproteinase inhibitor 1,10-phenanthroline and bivalent ion chelator EDTA. Then, IGFBP-4 proteolysis was performed, and the NT-IGFBP-4 concentration after the proteolytic reaction was measured as described in Section 4. Incubation both with EDTA (5 mM) and 1,10-phenanthroline (0.1 mM) caused a 70% decrease in initial PAPP-A-specific IGFBP-4 proteolysis in the case of both hiPSC-CMs and a 90% decrease in IGFBP-4 proteolysis by human recombinant homodimeric PAPP-A (Figure 2H,I).

Thus, the detection of NT-IGFBP-4 in the conditioned medium, the enhancement of NT-IGFBP-4 proteolytic fragment formation in the presence of IGF-II, and the inhibition of proteolytic activity by EDTA and 1,10-phenanthroline showed that IGFBP-4 proteolysis in the conditioned medium of hiPSC-CMs is PAPP-A-specific.

### 2.3. PAPP-A-Specific IGFBP-4 Cleavage Proceeds Both on the Cell Surface and in the Conditioned Medium of hiPSC-CMs

**Figure 3:**
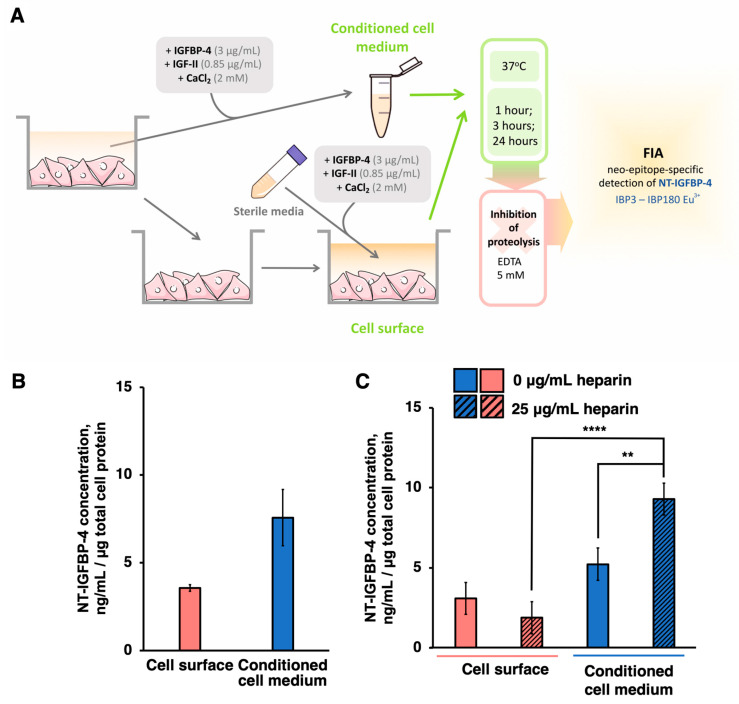
(**A**) Experimental design to study the PAPP-A-specific IGFBP-4 proteolysis cell localization. (**B**) NT-IGFBP-4 accumulation after 1 h of IGFBP-4 proteolytic reaction in conditioned medium and cell surface fractions of hiPSC-CMs derived from endo-IPS12 (*n* = 3). (**C**) Effect of heparin on PAPP-A-specific proteolytic cleavage of IGFBP-4 in the conditioned medium (blue) and cell surface fractions (red) of hiPSC-CMs after 1 h of IGFBP-4 proteolytic reaction (*n* = 4, **** *p* < 0.0001, ** *p* < 0.005). Clear bars correspond to NT-IGFBP-4 concentrations measured on the cell surface and in conditioned medium of control untreated hiPSC-CMs. Dashed bars correspond to the NT-IGFBP-4 concentration measured on the cell surface and in the conditioned medium of hiPSC-CMs pretreated with heparin (25 µg/mL). The obtained concentrations were normalized to total protein content measured by Bradford protein assay (see Section 4).

It was previously reported that, in several cell lines, proteolytically active PAPP-A adheres to the extracellular side of the membrane through GAG [6]. To test the distribution of proteolytically active PAPP-A in hiPSC-CMs, the conditioned medium from hiPSC-CMs derived from endo-IPS12 was obtained, and the fresh medium was added to the cells. Then, IGFBP-4 proteolysis was conducted both in the medium incubated with cells (cell surface proteolysis) and the conditioned medium (proteolysis in conditioned medium) for 1 h. Subsequently, the NT-IGFBP-4 concentration measurement was performed (Figure 3A). NT-IGFBP-4 was detected in both the cell-surface and conditioned media (Figure 3B). The NT-IGFBP-4 concentration in the conditioned medium was approximately 2 times higher than that in the cell surface. Thus, it can be concluded that PAPP-A-specific IGFBP-4 proteolysis exists both on the cell surface and in the conditioned medium of hiPSC-CMs with more efficient IGFBP-4 proteolytic degradation in the conditioned medium than on the cell surface.

Heparin was demonstrated to inhibit PAPP-A cell surface binding, as it competes with GAG for PAPP-A binding [6]. To check whether heparin removes PAPP-A from the cell surface, hiPSC-CMs were incubated with heparin (25 µg/mL) for 1 h, the medium was substituted with a fresh medium, and then IGFBP-4 proteolysis was performed, followed by an NT-IGFBP-4 concentration measurement on the cell surface and in conditioned medium fractions. The effect of heparin on the PAPP-A-specific IGFBP-4 proteolysis distribution between cell surface and cell secretome fractions is shown in Figure 3C. Preincubation with heparin resulted in a 1.5 times increase in NT-IGFBP-4 concentration in the conditioned medium after the IGFBP-4 proteolytic reaction compared to untreated hiPSC-CMs. The distribution of NT-IGFBP-4 formed as result of PAPP-A-specific IGFBP-4 proteolysis between the cell surface and cell secretome in untreated control cells corresponded to 37.2 ± 9.8% and 62.8 ± 8.5%, respectively (NT-IGFBP-4 concentration formed as result of PAPP-A-mediated proteolysis in both fractions was taken as 100%). After heparin treatment, the ratios changed to 17.3 ± 8.7% and 82.7 ± 6.1% in the corresponding fractions. At heparin concentration higher than 100 µg/mL the ratio of NT-IGFBP-4 between the cell surface and cell secretome fractions stayed at the 20.3 ± 5.3% and 80.7 ± 7.9% in corresponding fractions, suggesting that a further increase in heparin concentrations did not change the NT-IGFBP4-distribution between cell surface and secretome (*p* > 0.05 for both fractions). This finding meant that heparin removed PAPP-A from the cell membrane to the conditioned medium while approximately 10–20% of enzymatically active PAPP-A remained bound to the cell surface.

Taken together, our data show that proteolytically active PAPP-A is located both on the extracellular cell surface and in conditioned medium of hiPSC-CMs (Figure 3B,C).

## 3. Discussion

PAPP-A, IGFBP-4, and IGFBP-4 proteolytic fragments play a crucial role in the pathogenesis of CVDs, including coronary heart disease, acute coronary syndrome, and acute heart failure [18,19,20]. PAPP-A is also believed to be responsible for IGFBP-2, -4, and -5 proteolytic degradation and increasing the bioavailability of IGFs in various cell lines and tissues [4]. In several studies, IGFs, especially IGF-1, were applied as cardioprotectors in mouse models of myocardial injury, assuming that, in the normal heart, these factors could also sustain the cell viability and functioning of cardiomyocytes [2,3]. However, the mechanisms of IGF bioavailability and the role of PAPP-A in normal cardiac tissue have not yet been thoroughly studied.

We previously reported that PAPP-A-specific IGFBP-4 proteolysis occurred in the conditioned medium of rat neonatal cardiomyocytes [21]. Compared to rat neonatal cardiomyocytes, the obtained populations of hiPSC-CMs differentiated from two different iPS cell lines demonstrated higher purity (90% vs. 70%) and increased frequency of contractions (22 beats per minute and 28 beats per minute vs. 11 beats per minute). The concentrations of dimeric PAPP-A in the conditioned medium of hiPSC-CMs differentiated from both cell lines within 24 h of cultivation were determined at levels of up to 4.3 ± 1.33 ng/mL and 3.8 ± 1.1 ng/mL. These levels were similar to previously measured PAPP-A concentrations in the conditioned medium of vascular smooth muscle cells [16,35] and fibroblasts [11,34], although there are some limitations concerning different assays being applied for PAPP-A concentration measurements [17]. D’Elia et al. measured PAPP-A concentrations by FIA in the conditioned medium of cardiac progenitor cells (CaPCs) and detected that PAPP-A was homodimeric. Tetrameric proteolytically inactive PAPP-A was not detected. Surprisingly, the levels that we measured exceeded the PAPP-A concentration in the conditioned medium of cardiac progenitor cells (CaPCs) by 100 times. For the re-calculation of concentration, mIU/L were converted to ng/mL according to the 30 mIU/L–10 ng/mL ratio described by Qin et al. [38]. The drastic difference between PAPP-A concentrations in CaPC and hiPSC-CMs in a normal state may indicate the importance of PAPP-A-mediated cleavage of IGFBP-4, followed by the release of IGFs in adult cardiac tissue.

The IGFBP-4 proteolytic degradation in hiPSC-CM-conditioned media was proved to be PAPP-A-specific by different approaches. For the detection of NT-IGFBP-4, we applied a proteolytic neo-epitope-specific assay developed by our group [19,20,39,40] that detects the epitope forming as a result of specific IGFBP-4 proteolysis by PAPP-A. The observed concentrations of NT-IGFBP-4 in the conditioned medium of hiPSC-CMs derived from both cell lines were comparable to those that we measured earlier in the conditioned medium of rat neonatal cardiomyocytes [21]. The IGFBP-4 proteolytic cleavage by PAPP-A that we observed in hiPSC-CMs, as well as in the case of rat neonatal cardiomyocytes, was IGF-dependent. This outcome provides strong evidence that IGFBP-4 proteolysis in hiPSC-CMs is PAPP-A-specific. We also studied the inhibitory effects of EDTA and 1,10-phenanthroline on IGFBP-4 proteolytic degradation in the hiPSC-CM-conditioned medium compared to human recombinant homodimeric PAPP-A. Comparable effects of both EDTA and 1,10-phenanthroline on PAPP-A-specific IGFBP-4 proteolytic cleavage in hiPSC-CMs (70%) and human recombinant homodimeric PAPP-A (90%) provide additional evidence that IGFBP-4 proteolysis is PAPP-A-specific. In contrast, early studies of PAPP-A-specific IGFBP-4 inhibition measured by the western blot analysis of IGFBP-4 fragments showed that EDTA and 1,10-phenanthroline completely blocked PAPP-A proteolytic activity [41]. Data variations can be explained by the lower sensitivity of the western blot analysis applied in [41] compared to FIA, which we applied in the present study. The remaining PAPP-A proteolytic activity observed both in hiPSC-CMs and for recombinant PAPP-A could be related to additional factors in the cells’ secretome, stabilizing PAPP-A in the absence of Zn^2+^ and Ca^2+^.

Our results also demonstrate that proteolytically active PAPP-A localizes on both the cell surface and cell secretome of hiPSC-CMs, consistent with what Laursen et al. documented in other cell lines, including HEK293T, JAR-cells, BHK-21-cells, and human skin fibroblasts [6]. After heparin treatment, the level of PAPP-A-mediated IGFBP-4 proteolysis was increased in the cell secretome fraction, compared to the cell surface fraction. This finding suggests that heparin could interact with PAPP-A and detach it from GAG following its removal from the cell surface. Similar data were obtained by Laursen et al. in a PAPP-A overexpressed HEK293T cell model by flow cytometry analysis [6].

Together, these findings show that hiPSC-CMs derived from both iPS cell lines possess proteolytically active PAPP-A that specifically cleaves IGFBP-4 on the cell surface and in the extracellular space. Since IGFBP-4 proteolysis by PAPP-A leads to the release of IGFs, it can be assumed that cardiomyocytes, as well as other heart cells, such as fibroblasts and endothelial cells, could accumulate IGF in the extracellular space, and IGF might act as an autocrine and paracrine regulator in healthy myocardial tissue. This regulation might enable IGF interactions with specific IGF receptors and the activation of cardioprotective effects, including protection against ischemic myocardial damage [2,3]. Thus, the results of our study suggest that PAPP-A might play a role in sustaining cardiomyocyte viability and function in the healthy heart.

## 4. Materials and Methods

All the biochemical reagents applied were of analytical grade and were purchased from Sigma-Aldrich (Saint-Louis, MO, USA), ThermoFisher Scientific (Waltham, MA, USA), ICN (Costa Mesa, CA, USA), AppliChem (Darmstadt, Germany), Merck, (Rahway, NJ, USA), Dia-M (Moscow, Russia), and Bio-Rad (Hercules, CA, USA). The reagents for the cell culture experiments were purchased from HyClone (Logan, UT, USA), Sigma-Aldrich, Corning Costar (Corning, NY, USA), ThermoFisher Scientific, Stemcell Technologies (Vancouver, BC, Canada), and Paneco (Moscow, Russia). Secondary antibodies specific to the mouse Fc fragments conjugated to the Alexa 594 and DAPI, as well as phalloidin conjugated to the Alexa 488, were purchased from ThermoFisher Scientific. Monoclonal antibodies specific to the cardiac isoform of troponin T (clone 406), NT-IGFBP-4 (clones IBP3, IBP180), PAPP-A (clones 10E1, 10E2, 7A6, 5H9), and recombinant human IGFBP-4, NT-IGFBP-4, and PAPP-A were kindly provided by HyTest (Turku, Finland).

### 4.1. Differentiation of hiPSCs into Cardiomyocytes

The hiPSC line endo-iPS12 [42], established from HUVECs (human umbilical vein endothelium cells) from a healthy donor, and RG6L [43], established from human skin fibroblasts, were generated and described previously [42,43]. The cells were cultured in Matrigel (BD, Franklin Lakes, NJ, USA)-coated plates in mTeSR1 medium (Stemcell Technologies). The medium was changed daily. For the differentiation to cardiomyocytes, hiPSCs were detached with 0.05% trypsin and plated in 24-well plates at a density of 40,000 cells/cm^2^ in mTeSR1 medium supplemented with 5 μM Y-27632. Upon reaching 80–90% visible confluency, differentiation was started using a STEMdiff™ Ventricular Cardiomyocyte Differentiation Kit (Stemcell Technologies) according to the manufacturer’s protocol with modifications (differentiation day 0, d0). Briefly, for d0, d2, and d4, the medium was completely changed, and differentiation media A, B, and C were added, respectively. On d6 and d8, the media were completely changed, and differentiation medium C and maintenance medium were added. On d8, the first clusters of contracting cells were observed. On d10, the maintenance medium was changed to medium CDM3L, which consists of RPMI 1640 with no glucose (ThermoFisher Scientific, cat. no. 11879-020), 1× Glutamax, 500 μg/mL recombinant human albumin, and 213 μg/mL L-ascorbic acid supplemented with 4 mM L-lactic acid (Sigma-Aldrich). The medium was changed every other day. The CDM3L medium allowed for the metabolic selection of cardiomyocytes and contributed to a significant increase in the purity of the population. On d14, the medium was changed to the maintenance medium, and this medium was changed every other day. On d18, cardiomyocytes were subcultured 1:2–1:3 with 0.25% trypsin solution and plated in 24-well plates. Cells at passages 2–3 were used for experiments.

### 4.2. Immunocytochemistry

hiPSC-CMs were washed with phosphate-buffered saline (PBS) and then fixed in 4% paraformaldehyde and permeabilized in 0.1% Triton X-100, each for 15 min at RT. Subsequently, the cells were incubated in the presence of 1% fetal bovine serum (FBS) for 30 min to block nonspecific sorption of antibodies, followed by overnight treatment with primary antibodies specific to troponin T (clone 406, Hytest) at +4 °C. Next, the cells were incubated with Alexa-594-conjugated secondary antibodies specific to the mouse Fc fragments (ThermoFischer Scientific) for 1 h at RT. Incubations with Alexa-488-conjugated phalloidin and DAPI (both ThermoFischer Scientific) in the dilutions recommended by the manufacturer were also performed for 1 h at RT. All the incubation steps were followed by washing with PBS at RT. The immunochemical stained cells were visualized using an Olympus FV300 microscope (Tokyo, Japan).

### 4.3. Flow Cytometry

Cells were detached with trypsin–EDTA (0.25%) at 37 °C and 5% CO_2_. Then, Dulbecco’s Modified Eagle Medium (DMEM) containing 3% FBS was added to inhibit trypsin proteolytic activity. Cell suspensions were centrifuged at 300× *g* for 5 min at 25 °C, the supernatant was removed, and the cells were washed 3 times with PBS. Then, the cells were fixed in 4% paraformaldehyde for 30 min on ice and gently washed 3 times with cold PBS. Each sample was incubated on ice for 30 min with cold 80% ethanol, followed by washing 3 times with fluorescence activated cell sorting (FACS) buffer (PBS supplemented with 1% FBS). Next, the cells were incubated with IgG isotype control and antibodies for the human cardiac isoform of troponin T (clone 406) in FACS buffer overnight at +4 °C. Each sample was then incubated in FACS buffer with secondary anti-mouse polyclonal antibodies conjugated with Alexa-488 on ice for 30 min. The cells were rinsed twice with FACS buffer and then mixed with an equal volume of 200 ng/mL DAPI solution. Flow cytometry analyses were performed using an Novocyte Novosampler flow cytometer (ACEA Biosciences, San Diego, CA, USA).

### 4.4. Proteolytic Cleavage of IGFBP-4 in Cell-Conditioned Medium

Proteolysis of IGFBP-4 in the conditioned medium of cardiomyocytes was performed according to the protocol of Laursen et al. [44] with some modifications. The number of independently differentiated batches of cardiomyocytes (n) used in experiments varied from three to 11. For IGFBP-4 proteolysis, a 10× substrate mixture containing 3 μg/mL of recombinant human IGFBP-4, 0.85 μg/mL recombinant human IGF-II (Sigma Aldrich cat. no. SRP3070) and 2 mM CaCl_2_ was prepared in Tris-saline buffer (20 mM Tris-HCl, 150 mM NaCl, pH 7.4). In experiments in which IGFBP-4 proteolysis was performed in the absence of IGF, no IGF was added to the substrate mixture. The substrate mixture was incubated for 30 min at RT to form a complex of IGFBP-4 and IGF-II. Then, it was added to the conditioned medium of cardiomyocytes or to the sterile medium as a control, followed by incubation at +37 °C and 5% CO_2_ for 1, 3, or 24 h. At the end of incubation, the conditioned or sterile medium was removed from the wells or tubes, and the reaction was stopped by adding EDTA to a final concentration of 5 mM. The concentrations of NT-IGFBP-4 and PAPP-A were measured by a sandwich-type FIA as described below. The values of the concentrations of NT-IGFBP-4 and PAPP-A were normalized to the total amount of protein in lysates of the cells in each well measured by Bradford assay [45]. To study localization of PAPP-A-specific IGFBP-4 proteolysis, the media were obtained from cells and moved to a separate tube, fresh medium was added to the cells, and then IGFBP-4 proteolysis and NT-IGFBP-4 concentration measurements were performed in both fractions (Figure 3A). In the experiments with PAPP-A inhibition, hiPSC-CMs were pretreated with 5 mM EDTA and 0.1 mM 1,10-phenanthroline for 2 h or with water as a control. Then, IGFBP-4 proteolysis and the determination of the NT-IGFBP concentration were performed as previously described. In the heparin experiments, hiPSC-CMs were pretreated with 25 µg/mL heparin or water as a control for 2 h before IGFBP-4 proteolysis and NT-IGFBP-4 concentration measurements.

### 4.5. Detection of NT-IGFBP-4 and PAPP-A by Sandwich-Type FIA

The concentrations of NT-IGFBP-4 and PAPP-A in the conditioned media were determined by a sandwich-type FIA using antibody pairs IBP3–IBP180-Eu^3+^ (neo-epitope-specific, determining the concentration of NT-IGFBP-4), and 10E2–10E1-Eu^3+^ and 7A6–5H9-Eu^3+^ (determining the concentration of PAPP-A), as described by Konev et al. [39,40]. For FIA, the capture antibodies (20 μg/mL, 0.1 mL/well in PBS) were sorbed onto the surface of the wells of a 96-well polystyrene plate by shaking for 30 min at RT. The wells were then washed with 10 mM Tris-HCl, 150 mM NaCl, 0.025% Tween 20, and 0.05% NaN_3_ pH 7.8 (buffer A). Subsequently, 0.05 mL of the sample was diluted in the assay buffer (50 mM Tris-HCl buffer, 0.9% NaCl, 0.01% Tween 40, 0.5% bovine serum albumin, 0.05 % NaN_3_ pH 7.7), and 0.05 mL of detector antibodies (4 μg/mL) conjugated with a stable europium chelate (Eu^3+^) was added to each well. The plates were incubated for 30 min at RT with constant shaking. After washing with buffer A, 0.2 mL of fluorescent enhancement solution was added to the wells of the plate, and then the fluorescence was detected using a VICTOR X multilabel plate reader (PerkinElmer, Waltham, MA, USA).

### 4.6. Cell Lysate Preparation

To prepare cell lysates, hiPSC-CMs were washed 3 times with PBS, followed by the addition of 300 µL of lysis buffer (PBS with 0.1% Triton X-100, 20 µg/mL phenylmethylsulfonyl fluoride (PMSF), 3 µg/mL aprotinin, 1 µm pepstatin A). Then, the samples were subjected to ultrasonic treatment using Branson Sonifier (Branson Ultrasonics, Brookfield, CT, USA), frozen, and stored at −20 °C. The protein concentrations in cell lysates were determined using the Bradford protein assay, as described in [45]. The obtained values were utilized to normalize the PAPP-A and NT-IGFBP-4 concentrations.

### 4.7. Statistical Analysis

The Shapiro–Wilk test was performed to evaluate the sample distribution. We employed Student’s *t*-test and one-way analysis of variance (ANOVA) for normally distributed data, followed by post hoc analysis with Bonferroni’s adjustment for multiple comparisons. We employed the Mann–Whitney test and the Kruskal–Wallis test for non-normally distributed data, followed by Dunn’s post-hoc test. The data are presented as the mean ± standard deviation or the mean ± standard error of the mean. At *p* ≤ 0.05, differences were considered statistically significant.

## Data Availability

Not applicable.

## Supplementary Materials

The following supporting information can be downloaded at: https://www.mdpi.com/article/10.3390/ijms24098420/s1.

## Abbreviations

ANOVA—analysis of variance; CVD—cardiovascular disease; CaPC—cardiac progenitor cells; CT-IGFBP-4—C-terminal IGFBP-4 proteolytic fragment; cTnT—cardiac isoform of troponin T; DAPI—4′;6-diamidino-2-phenylindole; DMEM—Dulbecco’s Modified Eagle Medium; EDTA—ethylendiaminetetraacetic acid; FACS—fluorescence activated cell sorting; FBS—fetal bovine serum; FIA—fluoroimmune analysis; GAG—glucosaminoglycane; hiPSC-CMs—human induced pluripotent stem cell-derived cardiomyocytes; IGFs—insulin-like growth factors; IGF-I—insulin-like growth factor-II; IGF-II—insulin-like growth factor-II; IGFBP 1–6—insulin-like growth factor binding proteins 1-6; NT-IGFBP-4—N-terminal IGFBP-4 proteolytic fragment; PAPP-A—pregnancy associated plasma protein A; PBS—phosphate-buffered saline; PMSF—phenylmethylsulfonylfluoride; proMBP—proform of eosinophil major basic protein.
